# Sesame oleosins are minor allergens

**DOI:** 10.1186/s13601-019-0271-x

**Published:** 2019-06-28

**Authors:** Anna M. Ehlers, Madlen Rossnagel, Bettina Brix, Mark A. Blankestijn, Thuy-My Le, Waltraud Suer, Henny G. Otten, André C. Knulst

**Affiliations:** 1 Laboratory of Translational Immunology, University Medical Centre Utrecht, Utrecht University, Utrecht, The Netherlands; 2 Department of Dermatology and Allergology, University Medical Centre Utrecht, Utrecht University, Utrecht, The Netherlands; 3 EUROIMMUN AG, Lübeck, Germany

**Keywords:** Diagnostics, Food allergy, IgE hypersensitivity3 oleosins, Sesame

## Abstract

**Background:**

In daily practice, one-third of sesame allergic patients, confirmed by clinical history or food challenge, do not show any detectable specific IgE using current diagnostics. Currently used sesame extracts are water-based and therefore lacking hydrophobic proteins like oleosins. Oleosins, the stabilizer of lipid droplets in plants, are described as allergens in sesame, peanut and hazelnut. In this study, we examine the role of oleosins in sesame allergy and their potential cross-reactivity between sesame and (pea)nuts.

**Methods:**

Specific IgE and IgG sensitisation to native and heterologously expressed sesame components and oleosins from other nuts, free of seed storage proteins, was assessed by line blot and sera from 17 sesame allergic patients without detectable specific IgE sensitisation to sesame, and compared to 18 sesame allergic and 13 tolerant patients with specific IgE sensitisation to sesame.

**Results:**

Sesame allergic patients without sensitisation showed no specific IgE to the tested sesame oleosins or components. Low levels of specific IgE to sesame oleosins were detected in 17% of sesame allergic and 15% of tolerant patients with sIgE sensitisation. Oleosins were recognised by serum IgG from multiple patients confirming immune reactivity and excluding technical issues leading to lack of specific IgE-binding to oleosins.

**Conclusion:**

Sesame oleosins are minor allergens and appear to have no additonal value in diagnosing sesame allergy in adults based on sIgE and sIgG detection. There is a high need for additional diagnostic tools in those patients to minimize the number of required food challenges.

**Electronic supplementary material:**

The online version of this article (10.1186/s13601-019-0271-x) contains supplementary material, which is available to authorized users.

To the editor

Diagnosis of sesame allergy by measuring specific IgE (sIgE) is based on extracts or the major allergen: 2S albumin Ses i 1. However, this leads to false negative results in around 30% of sesame allergic patients, a high frequency compared to other food allergies [1]. To overcome this obstacle, we evaluated the role of oleosins in sesame allergy. Oleosins are oil-body stabilizing proteins and might be lacking in water-based extracts due to its hydrophobicity [2].

Adult sesame allergic and tolerant but sensitised patients who visited the outpatient clinic of the University Medical Centre Utrecht, The Netherlands, were retrospectively selected and allergy (n = 35) or tolerance (n = 13) was confirmed by food challenge or an experienced physician diagnosis. Sesame allergic patients were subdivided into patients without and with detectable sIgE (ImmunoCAP sesame extract ≥ 0.35 kU/L; without n = 17; with n = 18). Patient characteristics are described in Table 1. Sera with sIgE against native and heterologously expressed oleosins from different nuts and seeds acted as positive controls. Ethical approval was acquired from the biobank committee of the University Medical Centre Utrecht, number 18-428.

**Table 1:** Patient demography of included sesame allergic and tolerant patients; allergy was defined by food challenge or an experienced physician

	Group 1a (n = 17)^a^	Group 1b (n = 18)^a^	Group 2 (n = 13)^a^	*p* values
Age (median)	53 (29–85)	35 (27–51)	32 (27–58)	0.001
Sex female	14 (82%)	10 (56%)	10 (77%)	0.1865
Food challenge	8 (47%)	1 (6%)	1 (8%)	0.0034
Total IgE (kU/l)	295 (39 to > 5000)	4470 (1010 to > 5000)	> 5000 (518 to > 5000)	< 0.0001
*Symptoms* ^b^				
Mild (Müller 0)	6 (35%)	6 (33%)	N.A.	0.9693
Moderate (Müller 1 + 2)	6 (35%)	6 (33%)	N.A.	
Severe (Müller 3 + 4)	5 (29%)	6 (33%)	N.A.	
*IgE measurements* ^c^				
Sesame extract (n; median, range)	17 (0, 0–0.32 kU/L)	18 (4.5, 0.5–75 kU/L)	13 (3.9, 0.4–48 kU/L)	0.5338
Ses i 1 ISAC (n; median, range)	6 (0 ISU)	10 (2.9, 0–22.5 ISU)	3 (0, 0–6 ISU)	0.4336

^a^*G1a* sesame allergic patients without detectable sIgE sensitisation, *G1b* sesame allergic patients with sIgE sensitisation, *G2* sesame tolerant patients with sIgE sensitisation

^b^Symptom distribution of each group is shown in Additional file 3 :Figure S2

^c^ImmunoCAP and ISAC data were compared between group 1b and 2 since group 1a was selected by lacking sIgE sensitisation; CAP > 0.35 kU/L was considered as positive

Known sesame components and oleosins (from sesame, walnut, hazelnut, peanut and soy) with and without the hydrophobic domain based on the TMHMM model (prediction of transmembrane helices being not available for antibody binding) were heterologously expressed [3]. For comparisons to the native form, oil-body associated proteins (OAPs) were isolated from sesame, walnut and pecan using a modified previously described method with an additional hydrophobic interaction chromatography (HIC) instead of a preparative gel electrophoresis to separate traces of seed storage proteins with similar molecular masses [4]. To examine the absence of seed storage proteins, enclosed proteins were identified by mass spectrometry and detected by western blot with anti-human IgE-alkaline phosphatase (AP). Sensitisation to these components was deeply investigated by measuring sIgE and sIgG levels using line blots (EUROLINE, EUROIMMUN, Luebeck, Germany) according to manufacturer’s instructions. IgG subtypes were examined using an ELISA coated with different heterologously expressed oleosins (Ses i 4, Ses i Oleosin, Jug r Oleosin-1, Jug r Oleosin-2) and detected with anti-human IgG1-4 AP-conjugates. Detailed description of the methods is listed in Additional file 1.

Overall, the median age was 37 and allergic subjects suffered from typical symptoms being in line with an IgE-mediated hypersensitivity (characteristics are shown in Table 1). Gastro-intestinal symptoms were always accompanied by OAS or skin reactions (Additional file 2: Figure S1) and the allergic reactions took place within several minutes to half an hour after ingestion (data from 8/35), supporting the diagnosis ‘food allergy’.

The applied sesame OAPs fraction (pool of HIC-fractions 4 and 5) was free of seed storage protein traces as confirmed by western blot analysis with sera positive for these proteins from the study cohort (Fig. 1, part a). Before performing a HIC, these sera reacted with proteins around 15 kDa, similar molecular masses as oleosins. After the additional purification step, only the positive control (PC) still recognised proteins at this height. Additionally, no seed storage proteins but sesame oleosins were detected by mass spectrometry (Additional files 3 and 4: Figure S2 and Tables S1–S3).

**Fig. 1:**
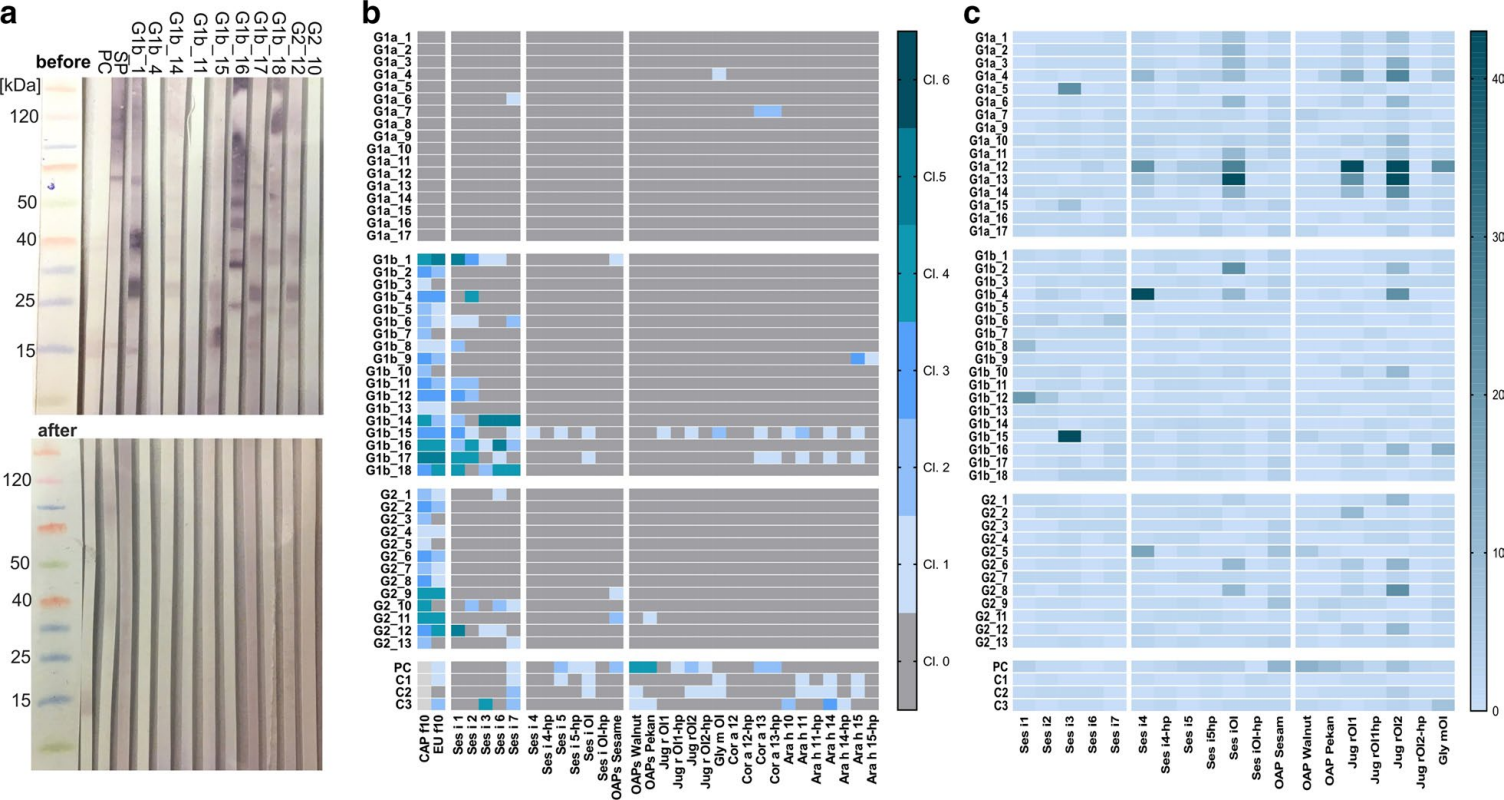
Western blot analysis of native sesame OAPs fraction to confirm the absence of seed storage protein traces (**a**) and the specific IgE (**b**) and IgG sensitisation pattern (**c**) of all included patients. **a** Western-Blot analysis of an intermediate purification step (before HIC) and of the final OAPs fraction (after HIC) with sera containing sIgE to sesame seed storage proteins and oleosins. Samples were separated by SDS-PAGE using a 4–12% Bis–Tris gel and blotted onto a nitrocellulose membrane. Bound IgE were detected by anti-human IgE antibodies conjugate labelled with alkaline phosphatase; PC is a oleosin-positive serum, SP is a sesame seed storage positive serum not used in this study, sera G1b_1, 4, 14, 11, 15–18, G2_12 and 10 are sera with specific IgE to sesame seed storage proteins and partly with slightly elevated sIgE levels to sesame oleosins. **b** IgE sensitisation pattern to sesame extract from ImmunoCAP and EUROLINE, sesame components and oleosins from sesame, walnut, soy, hazelnut and peanut expressed as EAST (Enzyme-Allergo-Sorbent Test classification)-classes and sorted by severity of symptoms to sesame, light grey: not determined. **c** IgG-sensitisation pattern to sesame components and oleosins from sesame, walnut, pecan and soy expressed as EUROLINE (EL)-intensities and sorted by severity of symptoms to sesame. *G1a* sesame allergic patients without detectable sIgE sensitisation, *G1b* sesame allergic patients with sIgE sensitisation, *G2* sesame tolerant patients with sIgE sensitisation, *PC* positive control selected by sIgE to sesame oleosins, *C1*-*3* positive controls selected by sIgE to peanut oleosins

Specific IgE-binding to sesame oleosins with levels above the detection limit (EAST-class 1–2) was detected in 17% of sensitised allergic and 15% of tolerant patients. However, none of the non-sensitised sesame allergic patients showed sIgE-binding neither to sesame oleosins nor to any other sesame components, except one serum showing low sIgE level to the 11S globulin Ses i 7 (G1a_6). Control sera, selected for sIgE binding to sesame (PC) or peanut oleosins (C1-3), showed sIgE binding to oleosins from different sources, confirming the binding capacity of heterologously expressed and native oleosins. Specific IgE-binding to oleosins in sensitised allergic patients was accompanied by recognising other sesame components, especially Ses i 1 and 2 whilst in tolerant patients was not (Fig. 1, part b and c). Patients showing IgE reactivity to heterologously expressed sesame oleosins were co-sensitised to heterologously expressed oleosins from walnut, hazelnut and peanut, indicating potential cross-reactivity (G1b_15, G1b_17). All oleosins expressed as full-length variant were recognised at least by one serum, except Cor a 12. In case of Cor a 13, the full-length and the hp-variant of Cor a 13 was recognised in parallel (G1b_17, PC).

Although oleosins were infrequently recognised by sIgE, they were bound by serum IgG from multiple patients across all groups confirming immune reactivity and excluding technical issues leading to lack of specific IgE-binding. Depletion of IgG by protein G columns in these sera did not result in sIgE binding (Additional file 5: Figure S3), excluding competition between IgG and IgE. Specific IgG was mainly detected for Ses i 4, Ses i Ol, Jug r Ol-1 and Jug r Ol-2 and significantly increased in sesame allergic patients without detectable sIgE sensitisation compared with the other groups (Ses i 4: G1a vs. G1b, *p *= 0.0014; Ses i Ol: G1a vs. G2, *p *= 0.03; Jug r Ol-2: G1a vs. G1b, *p *= 0.0049). The most prevalent subtype recognising oleosins was IgG1, while IgG2, IgG3 and IgG4 levels were dependent on the oleosin of interest (Additional file 6 and 7: Figures S4 and S5).

Up to 30% of sesame allergic patients, including those suffering from severe reactions, cannot be diagnosed by commercially available diagnostic tests [1, 5]. Launched aqueous sesame extracts do not contain hydrophobic proteins like oleosins. We demonstrated that oleosins were clearly recognized in 17% of sensitised sesame allergic patients, but did not have any additional diagnostic value compared to sesame extract and Ses i 1, especially in patients without detectable IgE sensitisation. Contrary to our findings, 90% recognition in sesame allergic patients (31% without sIgE to sesame extract) was reported previously [6]. Moreover, OAPs reactivity was detected in 36% of hazelnut allergic patients and therefore considered relevant diagnostic markers, particularly in patients without detectable sIgE [7]. This discrepancy might be explained by the absence of seed storage proteins or fragments thereof [8] in our native preparation accomplished by HIC which was confirmed by western blot analysis and mass spectrometry.

Oleosins were recognised by sIgE levels slightly above the detection limit (EAST-class 1–2). This is in line with a hazelnut study across Europe showing a prevalence of 20% for the hazelnut oleosin Cor a 12, but low sIgE titre up to 1 kU/l (≙ EAST-class 2) [9]. In our test system, even lower sIgE titres might be explained by competition between different oleosins coated on the same line blot.

In conclusion, sesame oleosins are minor allergens and appear to have no additional value in diagnosing sesame allergy in adults based on sIgE and sIgG detection. We propose a prospective study to evaluate the diagnostic value of direct basophil activation tests due to the high need for additional diagnostic tools in those patients.

## Additional files

**Additional file 1.** Supplementary methods. Detailed description of the methods.

**Additional file 2: Figure S1.** Distinct symptom distribution between sesame allergic patients with and without detectable sIgE sensitisation. Sesame allergic patients without detectable sIgE sensitisation (G1a) showed more often skin related reactions (Mueller 1) compared to patients with sensitisation (G1b). The other way around, patients of G1b showed more often gastro-intestinal symptoms (Mueller 2) and cardiovascular reactions (Mueller 4).

**Additional file 3: Figure S2.** Gel images for mass spectrometry analyses of OAPs fractions. Gel images of elution fractions of OAPs from sesame, walnut and pecan nut after hydrophobic interaction chromatography used for mass spectrometry analyses.

**Additional file 4: Table S1-S3.** Enclosed proteins in native OAPs fractions. The native OAPs fractions were analysed by mass spectrometry. Table 1-3 show the identified proteins in those fractions.

**Additional file 5: Figure S3.** IgG depletion does not influence the recognition of sesame oleosins by sIgE. After IgG depletion, sera with high sIgG levels to oleosins (G1a_4, G1a_12, G1a_13 and G2_8) showed no increase in sIgE levels to sesame components or oleosins while sera with low sIgG levels but IgE sensitisation to sesame (G1b_1, G1b_15, G1b_17, G2_9 and G2_11) showed scarcely a decrease in sIgE levels, confirming no depletion of IgE during IgG depletion. One serum positive for sesame oleosins, G1b_15, displayed an increase in sIgE levels to oleosins although no sIgG to these components were detected.

**Additional file 6: Figure S4.** Subtype analysis of sera with elevated sIgG levels for Ses i 4, Ses i Ol, Jug r Ol-1 and Jug r Ol-2. Detection of the IgG subtype bound to Ses i 4, Ses i Oleosin, Jug r Oleosin-1 and Jug r Oleosin-2 from serum with specific IgG to oleosins (EUROLINE-intensities > 8); Scatter-blot with the measured OD value ratios (sample/negative value [NV]) divided by subtype for each oleosin. Horizontal lines mark the mean value. Green: Group 1a - sesame allergic patients without detectable sIgE sensitisation; Red: Group 1b - sesame allergic patients with sIgE sensitisation; Black: Group 2 - sesame tolerant patients with sIgE sensitisation.

**Additional file 7: Figure S5.** Comparison of IgG subtype between allergic and tolerant patients. Detection of the IgG subtype bound to Ses i 4, Ses i Oleosin, Jug r Oleosin-1 and Jug r Oleosin-2 from serum with specific IgG to oleosins (EUROLINE-intensities > 8); the data were separated by allergic and tolerant patients, IgG subtype and protein (oleosin).

## Data Availability

All data generated or analysed during this study are included in this published article and its Additional information files.

## Abbreviations

EAST: Enzyme-Allergo-Sorbent Test
EL: EUROLINE
HIC: hydrophobic interaction chromatography
hp-variant: heterologous expressed oleosin without hydrophobic domain
NCBI: National Centre of Biotechnology Information
OAPs: oil-body associated proteins
sIgE: specific IgE
SPT: skin prick test
TMHMM: transmembrane prediction by hidden Markov model
