# Supplementary material for: Sesame oleosins are minor allergens
Source: Clin Transl Allergy. 2019 Jun 28;9:32. doi: 10.1186/s13601-019-0271-x (PMC6599271; doi:10.1186/s13601-019-0271-x)
Supplement: Supplementary file 1 — Additional file 1. Supplementary methods. Detailed description of the methods. [file 13601_2019_271_MOESM1_ESM.docx]

**Methods**

*Patient selection*

Sesame allergic and tolerant patients who visited the outpatient clinic of the University Medical Centre Utrecht, The Netherlands, between 2012 and 2017 were retrospectively selected. The selected patients were first divided into one of two groups regarding allergy (n=35) or tolerance (n=13) confirmed by food challenge or an experienced physician. Allergic patients were subdivided into patients without (n=17) and with (n=18) detectable sIgE sensitisation (ImmunoCAP sesame extract ≥ 0.35 kU/L). Tolerant patients without detectable sIgE sensitisation were excluded. Positive controls were sera with sIgE against native and heterologously expressed oleosins from different nuts and seeds. Ethical approval was acquired from the biobank committee of the University Medical Centre Utrecht under the number 18-428.

*Purification of oil-body associated proteins*

Oil-body associated proteins (OAPs) were isolated from sesame, walnut and pecan nut as described previously with the following modifications (1-3): Sesame seeds were ground 1:2 w/v in 50 mM NaH_2_PO_4_ buffer pH 7.4 containing 1 mM EDTA, 10 mM KCl, 2 mM DTT and 20% w/v sucrose (grinding buffer) using a blender. The ground sesame seeds were filtered over a gauze and layered 1:1 v/v with flotation buffer (50 mM NaH_2_PO_4_, 1 mM EDTA, 10 mM KCl, 2 mM DTT, pH 7.4). The gradient was centrifuged for 30 min at 4 °C using a swing-bucket rotor at 16.000 x g. After centrifugation, a white fat pad was formed on top of the gradient. After resuspension of the fat pad in urea buffer (50 mM NaH_2_PO_4_, 9 M urea, pH 7.4), the suspension was incubated for 15 min at room temperature, layered 1:1 v/v with 50 mM NaH_2_PO_4_ buffer pH 7.4 and centrifuged under the same conditions. This step was repeated twice, and the resulting fat pad was resuspended in grinding buffer containing 0,1 % Tween-20 and layered 1:1 v/v with 50 mM NaH_2_PO_4_ buffer pH 7.4. After centrifugation, the fat pad was resuspended in grinding buffer containing 2 M NaCl and layered with flotation buffer containing 2 M NaCl. After resuspension of the fat pad in grinding buffer, proteins were precipitated with a fourfold volume of ice-cold acetone and washed twice. After air-drying, the pellet was solubilised in a detergent-containing buffer and dialyzed against a 50 mM TRIS buffer, pH 7.5. To separate traces of seed storage proteins, the protein solution was applied on a hydrophobic interaction chromatography column.

*Heterologous expression and purification of recombinant allergens*

Sesame components (Ses i 1, 2, 3, 6 and 7) and oleosins (sesame, walnut, hazelnut and peanut) were expressed as fusion proteins with N-terminal His(6x) in E. coli as previously described (4, 5). The hp-variants of oleosins were expressed without the hydrophobic part determined by using the TMHMM model (6). All heterologous expressed proteins were purified by immobilised metal ion chromatography under denaturing conditions. Full-length oleosins were dialyzed against citrate buffer pH 5.5 for applying on the line blot.

*Mass spectrometry*

Enclosed proteins of the OAPs fractions from sesame, walnut and pecan nut were identified by mass spectrometry. Reduced (0.02 M DTT) and alkylated (0.06 M iodoacetamide) proteins were separated in a 4-12% polyacrylamide gel stained with colloidal Coomassie. After excising and achromatising, these proteins were digested in-gel by trypsin for 3 hours at 37 °C. The resulting peptides were subsequently extracted and spotted with α-cyano-4-hydroxycinnamic acid onto a MTP Anchor Chip 384 TF target (‎Bruker, Billerica, Massachusetts‎, US). Spotted peptides were measured by matrix-assisted laser desorption/ionisation-time of flight/TOF mass spectrometry (MALDI-TOF/TOF) using an Autoflex III smartbeam TOF/TOF200 System (Bruker, Billerica, Massachusetts‎, US) combined with the flexControl 3.4 software. MS spectra for peptide mass fingerprinting (PMF) were acquired in a positive ion reflector mode with 6000 shots ranging from 600 to 4.000 Da. For spectra calibration, external commercially available Peptide Calibration Standard II was processed with flexAnalysis 3.4 and the resulting peak lists were examined with BioTools 3.2. MS spectra for protein identification were analysed by submitting them to the MASCOT search engine MASCOT Server 2.3 (Matrix Science, London, U.K.) searching against the NCBI database (2016/12/19) including 33.467 proteins from *Sesamum indicum,* 56.036 proteins from *Juglans regia* and 278 proteins from *Carya illinoinensis*. Search parameters were set as follows: mass tolerance of 80 ppm, acceptance of one missed trypsin cleavage site, carbamidomethylation of cysteine residues as fixed modification, and oxidation of methionine residues as variable modification. For evaluation of protein hits, significance was defined as p < 0.05. PMF hits were confirmed by selecting two to five peptides of each identified protein with the WARP feedback mechanism of BioTools for MS/MS measurements. Recording of parent and fragment masses were performed with 400 and 1,000 shots, respectively. Resulted spectra were processed and analysed as described above with a fragment mass tolerance of 0.7 Da. These measurements were performed twice with different preparations in duplicates.

*Line Blot*

Sensitisation was defined by measuring sIgE and sIgG levels using a line blot (EUROLINE, EUROIMMUN, Luebeck, Germany) according manufacturer’s instructions. For IgE detection, line blots were incubated overnight with 1 ml diluted serum (1:11 in universal buffer), some of them IgG-depleted (Protein G columns, GE Healthcare, Buckinghamshire, Great Britain), at room temperature on a rocket shaker. For detection of sIgG levels, 0.51 ml diluted serum (1:51 in universal buffer) was incubated for one hour at room temperature. Bound serum antibodies were detected with anti-human IgE or IgG alkaline phosphatase-coupled conjugate and visualised by adding nitro-blue tetrazolium/5-bromo-4-chloro-3’-indolyphosphate substrate. The binding of antibodies was evaluated using the EUROLINEScan software. For IgE measurement, band intensities were reported as intensity levels or classes, leaned on the Enzyme-Allergo-Sorbent Test (EAST) classification (19). EUROLINE intensities (EL-intensities) of 3 or greater were considered as positive, corresponding to classes from 1 to 6. For IgG measurement, band intensities smaller than 8 were considered as negative. These measurements were performed once.

*Total IgE levels*

Total IgE levels were determined using the ELISA technique (EUROIMMUN, Lübeck, Germany). In brief, 100 µl of diluted serum (1:10 in universal buffer) were applied per well and incubated for 30 min at room temperature. Bound IgE was detected by applying anti-IgE horse radish peroxidase-coupled conjugate and visualised by adding tetramethylbenzidine. Optical intensities read at 450 nm were evaluated with a calibration curve and converted into IU/ml. These measurements were performed once in duplicates.

*IgG subtype determination*

Microtiter plates were coated with heterologously expressed full-length oleosins (9 µg/mL in PBS, pH 7.5) over night at 4 °C. After blocking, 100 µl of diluted serum (1:25 in blocking buffer) was applied in duplicates and allowed to react for 1 hour at room temperature on an orbital shaker. Bound antibodies were detected using anti-human IgG1-4 AP-conjugates and stained with p-Nitrophenyl Phosphate Disodium salt (Thermofisher Scientific, Waltham, MA, USA). The optical density (OD) was read at 405 nm using an automated spectrophotometer (iMark, Bio-Rad, Hercules, CA, USA). OD values of each IgG subtype measured for sera previously considered as positive (line blot) were divided by the OD value of the negative control. These measurements were performed twice in duplicates.

*Data analysis*

The baseline data were statistically analysed using one-way ANOVA or Mann-Whitney-U-test for continuous data and Fisher’s exact test for categorical data. For comparison of sIgG levels between the patient groups, Kruskal–Wallis and Dunn's post-hoc tests were used. Statistical evaluation was performed with GraphPad Prism 7 (GraphPad Software, La Jolla, CA, USA) and SPSS Statistics 21 (IBM Corporation, Armonk, NY, USA). P values ≤ 0.05 were considered as statistically significant.

**References**

1. Zuidmeer-Jongejan L, Fernández-Rivas M, Winter MGT, Akkerdaas JH, Summers C, Lebens A, et al. Oil body-associated hazelnut allergens including oleosins are underpresented in diagnostic extracts but associated with severe symptoms. *Clin Trans Allergy*. 2014;4.

2. Schwager C, Kull S, Krause S, Schocker F, Petersen A, Becker WM, et al. Development of a novel strategy to isolate lipophilic allergens (oleosins) from peanuts. *PLoS One*. 2015;10.

3. Tzen JT, Peng CC, Cheng DJ, Chen EC, Chiu JM. A new method for seed oil body purification and examination of oil body integrity following germination. *J Biochem.* 1997;121:762-8.

4. Hochuli E, Bannwarth W, Döbeli H, Gentz R, Stüber D. Genetic Approach to Facilitate Purification of Recombinant Proteins with a Novel Metal Chelate Adsorbent. *Bio/Technology*. 1988;6:1321-5.

5. Trigoso YD, Evans RC, Karsten WE, Chooback L. Cloning, Expression, and Purification of Histidine-Tagged *Escherichia coli* Dihydrodipicolinate Reductase. *PLoS One*. 2016;11:e0146525.
