# Supplementary figures and images for: Sesame oleosins are minor allergens
Source: Clin Transl Allergy. 2019 Jun 28;9:32. doi: 10.1186/s13601-019-0271-x (PMC6599271; doi:10.1186/s13601-019-0271-x)

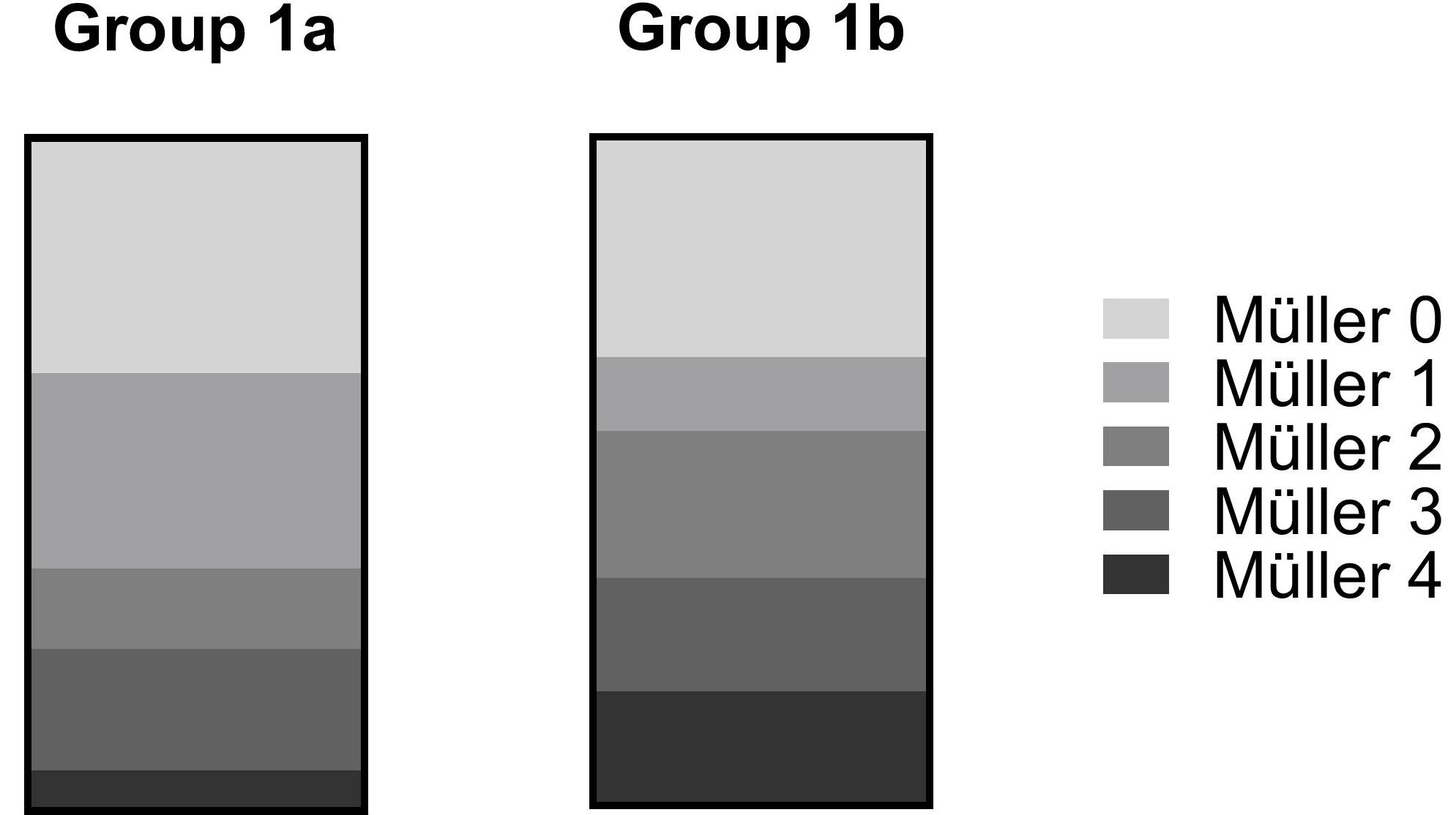

Supplement: Supplementary file 2 — Additional file 2: Figure S1. Distinct symptom distribution between sesame allergic patients with and without detectable sIgE sensitisation. Sesame allergic patients without detectable sIgE sensitisation (G1a) showed more often skin related reactions (Mueller 1) compared to patients with sensitisation (G1b). The other way around, patients of G1b showed more often gastro-intestinal symptoms (Mueller 2) and cardiovascular reactions (Mueller 4). [file 13601_2019_271_MOESM2_ESM.jpg]

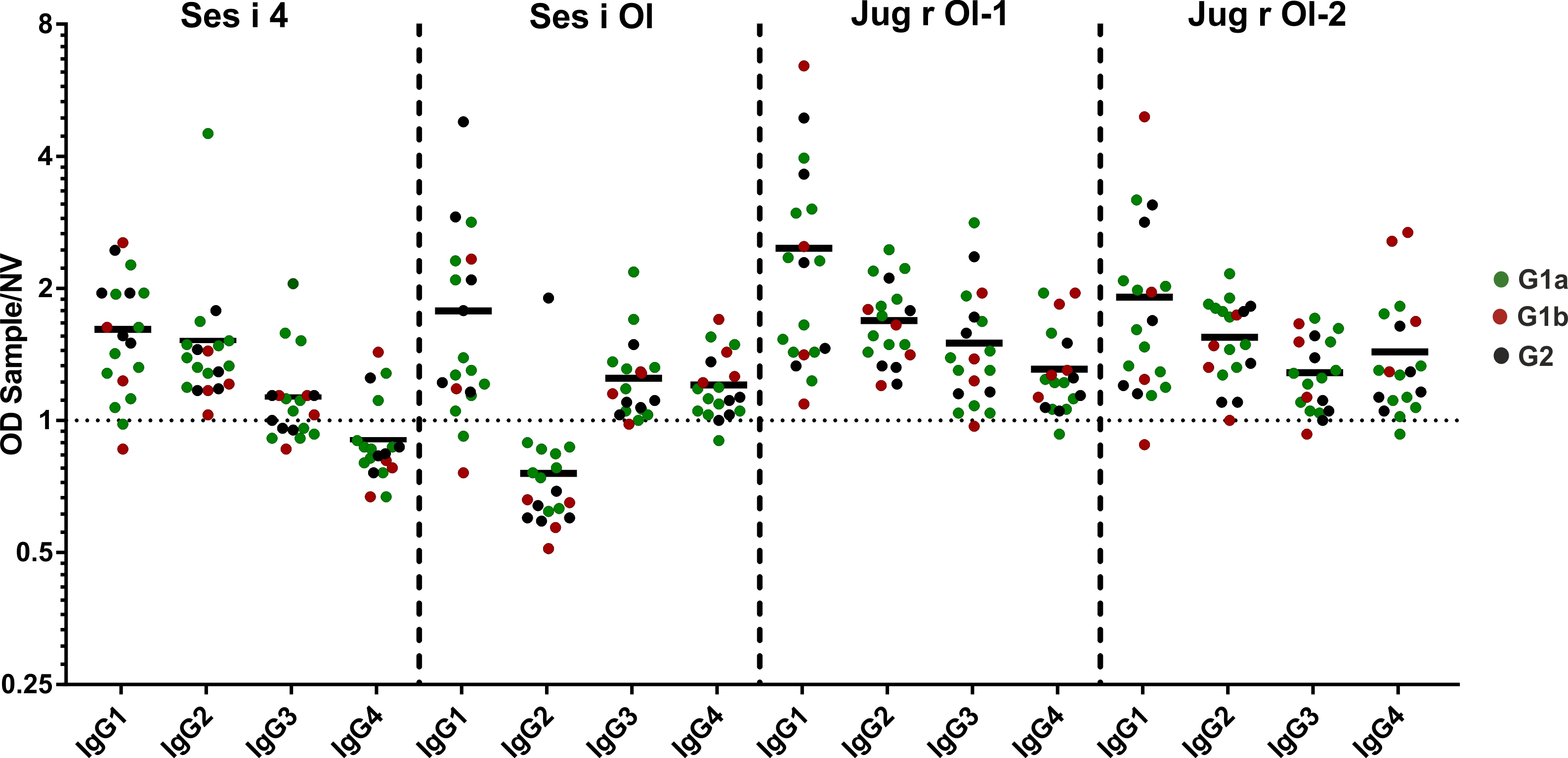

Supplement: Supplementary file 6 — Additional file 6: Figure S4. Subtype analysis of sera with elevated sIgG levels for Ses i 4, Ses i Ol, Jug r Ol-1 and Jug r Ol-2. Detection of the IgG subtype bound to Ses i 4, Ses i Oleosin, Jug r Oleosin-1 and Jug r Oleosin-2 from serum with specific IgG to oleosins (EUROLINE-intensities > 8); Scatter-blot with the measured OD value ratios (sample/negative value [NV]) divided by subtype for each oleosin. Horizontal lines mark the mean value. Green: Group 1a - sesame allergic patients without detectable sIgE sensitisation; Red: Group 1b - sesame allergic patients with sIgE sensitisation; Black: Group 2 - sesame tolerant patients with sIgE sensitisation. [file 13601_2019_271_MOESM6_ESM.jpg]
