# Supplementary material for: Sesame oleosins are minor allergens
Source: Clin Transl Allergy. 2019 Jun 28;9:32. doi: 10.1186/s13601-019-0271-x (PMC6599271; doi:10.1186/s13601-019-0271-x)
Supplement: Supplementary file 3 — Additional file 3: Figure S2. Gel images for mass spectrometry analyses of OAPs fractions. Gel images of elution fractions of OAPs from sesame, walnut and pecan nut after hydrophobic interaction chromatography used for mass spectrometry analyses. [file 13601_2019_271_MOESM3_ESM.pdf]

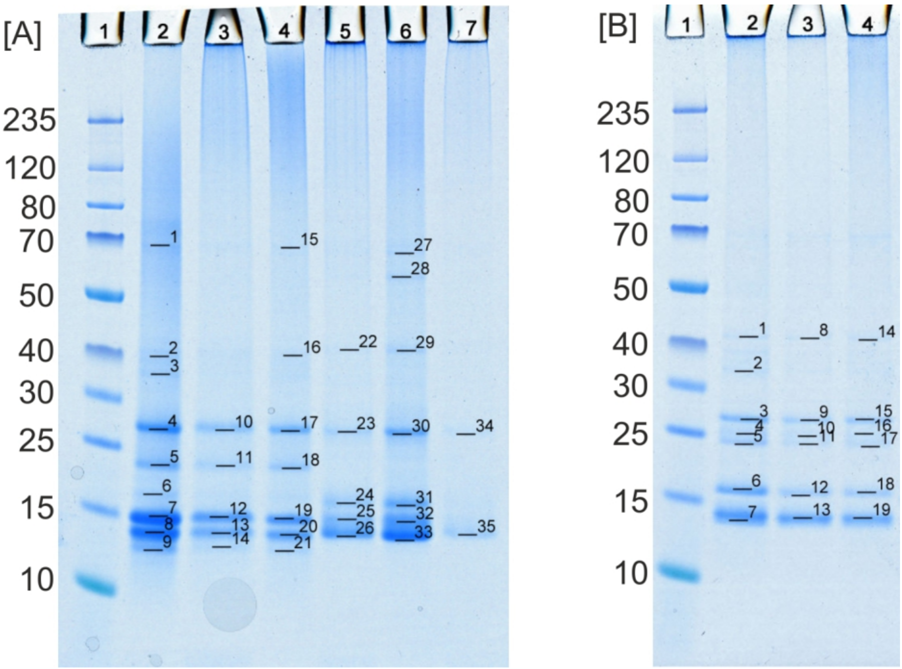

[A]
1 - Marker
2 - OAPs sesame HIC-fraction 3
3 - OAPs sesame HIC-fraction 4
4 - OAPs sesame HIC-fraction 5
5 - OAPs walnut HIC-fraction 3
6 - OAPs walnut HIC-fraction 4
7 - OAPs walnut HIC-fraction 5

[B]
1 - Marker
2 - OAPs pecan nut HIC-fraction 3
3 - OAPs pecan nut HIC-fraction 4
4 - OAPs pecan nut HIC-fraction 5

Figure S2: Enclosed proteins of the native OAPs fractions from sesame, walnut and pecan nut were identified by mass spectrometry. The proteins were seperated by gel electrophoresis using 4-12% bis-tris gels and the bands were excised and in-gel digested with trypsin.
[A] Elution fractions of hydrophobic interaction chromatography (HIC) from sesame (line 2-4) and walnut (line 5-7) OAPs
[B] Elution fractions of hydrophobic interaction chromatography (HIC) from pecan nut OAPs (line 2-4)
