# Supplementary material for: Sesame oleosins are minor allergens
Source: Clin Transl Allergy. 2019 Jun 28;9:32. doi: 10.1186/s13601-019-0271-x (PMC6599271; doi:10.1186/s13601-019-0271-x)
Supplement: Supplementary file 4 — Additional file 4: Table S1-S3. Enclosed proteins in native OAPs fractions. The native OAPs fractions were analysed by mass spectrometry. Table 1-3 show the identified proteins in those fractions. [file 13601_2019_271_MOESM4_ESM.pdf]

Table S1: The native sesame OAPs fractions was analysed by mass spectrometry using peptide mass fingerprint. The table shows the identified proteins in this fraction.

Band	Protein *(Accession number)*	Cut-Off	Protein Score	Sequence coverage [%]	Molecular Mass [kDa]
**Sesame OAPs: HIC-Elution-Fraction 3**					
1	uncharacterized protein LOC105177100 *(NP_001292915.1)*	49	45	10	17,4
	15 kDa oleosin *(AAD42942.1)*	49	23	6	15,2
	PREDICTED: oleosin 1-like *(XP_011076526.1)*	49	23	4	24,0
2	11-beta-hydroxysteroid dehydrogenase-like 5 *(NP_001291330.1)*	79/50	169/55	54/4	41,3
3	11-beta-hydroxysteroid dehydrogenase 1B-like *(NP_001291322.1)*	79/50	125/31	50/2	39,7
	11-beta-hydroxysteroid dehydrogenase-like 5 *(NP_001291330.1)*	79/50	87/20	41/4	41,3
4	peroxygenase *(NP_001291323.1)*	79/50	76/115	40/23	27,8
5	PREDICTED: legumin B-like *(XP_011083024.1)*	79/50	93/59	35/8	52,1
	Peroxygenase *(NP_001291323.1)*	79/50	80/81	32/10	27,8
	11S globulin precursor isoform 4 *(ABB60055.1)*	79/50	80/59	32/8	53,0
6	PREDICTED: legumin B-like *(XP_011083024.1)*	79	72	33	52,1
	11S globulin precursor isoform 4 *(ABB60055.1)*	79	60	28	53,0
	15 kDa oleosin *(AAD42942.1)*	50	22	6	15,2
	PREDICTED: oleosin 1-like *(XP_011076526.1)*	50	22	4	24,0
7	15 kDa oleosin *(AAD42942.1)*	50	61	6	15,2
	PREDICTED: oleosin 1-like *(XP_011076526.1)*	50	61	4	24,0
8	uncharacterized protein LOC105177100 *(NP_001292915.1)*	51	43	10	17,4
9	uncharacterized protein LOC105177100 *(NP_001292915.1)*	49	40	10	17,4
	15 kDa oleosin *(AAD42942.1)*	49	35	6	15,2
	PREDICTED: oleosin 1-like *(XP_011076526.1)*	49	35	4	24,0
	PREDICTED: oleosin 16.4 kDa *(XP_011097414.1)*	49	20	10	17,4

Sesame OAPs: HIC-Elution-Fraction 4

10	Peroxygenase *(NP_001291323.1)*	79/49	108/27	41/8	27,8
11	Peroxygenase *(NP_001291323.1)*	51	81	10	27,8
**12**	uncharacterized protein LOC105177100 *(NP_001292915.1)*	79	57	39	17,4
	15 kDa oleosin *(AAD42942.1)*	50	47	6	15,2
	PREDICTED: oleosin 1-like *(XP_011076526.1)*	50	47	4	24,0
**13**	uncharacterized protein LOC105177100 *(NP_001292915.1)*	51	24	10	17,4
	PREDICTED: oleosin 1-like *(XP_011081346.1)*	51	22	7	14,8
	15 kDa oleosin *(AAD42942.1)*	51	21	6	15,2
	PREDICTED: oleosin 1-like *(XP_011076526.1)*	51	21	4	24,0
**14**	PREDICTED: DDB1- and CUL4-associated factor 8 *(XP_011089371.1)*	79	58	22	54,1
	uncharacterized protein LOC105177100 *(NP_001292915.1)*	49	15	10	17,4

Sesame OAPs: HIC-Elution-Fraction 5

**15**	-	-	-	-	-
**16**	11-beta-hydroxysteroid dehydrogenase-like 5 *(NP_001291330.1)*	79/50	190/70	53/7	41,3
**17**	Peroxygenase *(NP_001291323.1)*	79/50	119/73	53/13	27,8
**18**	Peroxygenase *(NP_001291323.1)*	79/50	80/63	27/10	27,8
**19**	uncharacterized protein LOC105177100 *(NP_001292915.1)*	50	67	10	17,4
	15 kDa oleosin *(AAD42942.1)*	50	52	6	15,2
	PREDICTED: oleosin 1-like *(XP_011076526.1)*	50	52	4	24,0
**20**	PREDICTED: oleosin 1-like *(XP_011081346.1)*	51	46	7	14,8
	uncharacterized protein LOC105177100 *(NP_001292915.1)*	51	43	10	17,4
**21**	PREDICTED: DDB1- and CUL4-associated factor 8 *(XP_011089371.1)*	79	91	36	54,1
	uncharacterized protein LOC105177100 *(NP_001292915.1)*	49	17	10	17,4

Cut-off, protein-score and sequence coverage are highlighted in green if the results were obtained from MS/MS experiments

Table S2: The native walnut OAPs fractions was analysed by mass spectrometry using peptide mass fingerprint. The table shows the identified proteins in this fraction.

Band	Protein (Accession number)	Cut-Off	Protein Score	Sequence coverage [%]	Molecular Mass [kDa]
**Walnut OAPs: HIC-Elution-Fraction 3**					
22	-	-	-	-	-
23	-	-	-	-	-
24	-	-	-	-	-
25	-	-	-	-	-
26	Oleosin *(AET74077.1)*	79	40	38	14,7
**Walnut OAPs: HIC-Elution-Fraction 4**					
27	-	-	-	-	-
28	-	-	-	-	-
29	-	-	-	-	-
30	-	-	-	-	-
31	-	-	-	-	-
32	-	-	-	-	-
33	Oleosin *(AET74077.1)*	79	48	38	14,7
**Walnut OAPs: HIC-Elution-Fraction 5**					
34	-	-	-	-	-
35	Oleosin *(AET74077.1)*	79	53	35	14,7

Cut-off, protein-score and sequence coverage are highlighted in green if the results were obtained from MS/MS experiments

Table S3: The native pecan nut OAPs fractions was analysed by mass spectrometry using peptide mass fingerprint. The table shows the identified proteins in this fraction.

Band	Protein (Accession number)	Cut-Off	Protein Score	Sequence coverage [%]	Molecular Mass [kDa]
**Pecan nut OAPs: HIC-Elution-Fraction 3**					
1	-	-	-	-	-
2	-	-	-	-	-
3	-	-	-	-	-
4	-	-	-	-	-
5	-	-	-	-	-
6	-	-	-	-	-
7	-	-	-	-	-
**Pecan nut OAPs: HIC-Elution-Fraction 4**					
8	-	-	-	-	-
9	-	-	-	-	-
10	-	-	-	-	-
11	-	-	-	-	-
12	-	-	-	-	-
13	-	-	-	-	-
**Pecan nut OAPs: HIC-Elution-Fraction 5**					
14	-	-	-	-	-
15	-	-	-	-	-
16	-	-	-	-	-
17	-	-	-	-	-
18	-	-	-	-	-
19	-	-	-	-	-

Cut-off, protein-score and sequence coverage are highlighted in green if the results were obtained from MS/MS experiments
