# Supplementary material for: Sesame oleosins are minor allergens
Source: Clin Transl Allergy. 2019 Jun 28;9:32. doi: 10.1186/s13601-019-0271-x (PMC6599271; doi:10.1186/s13601-019-0271-x)
Supplement: Supplementary file 5 — Additional file 5: Figure S3. IgG depletion does not influence the recognition of sesame oleosins by sIgE. After IgG depletion, sera with high sIgG levels to oleosins (G1a_4, G1a_12, G1a_13 and G2_8) showed no increase in sIgE levels to sesame components or oleosins while sera with low sIgG levels but IgE sensitisation to sesame (G1b_1, G1b_15, G1b_17, G2_9 and G2_11) showed scarcely a decrease in sIgE levels, confirming no depletion of IgE during IgG depletion. One serum positive for sesame oleosins, G1b_15, displayed an increase in sIgE levels to oleosins although no sIgG to these components were detected. [file 13601_2019_271_MOESM5_ESM.pdf]

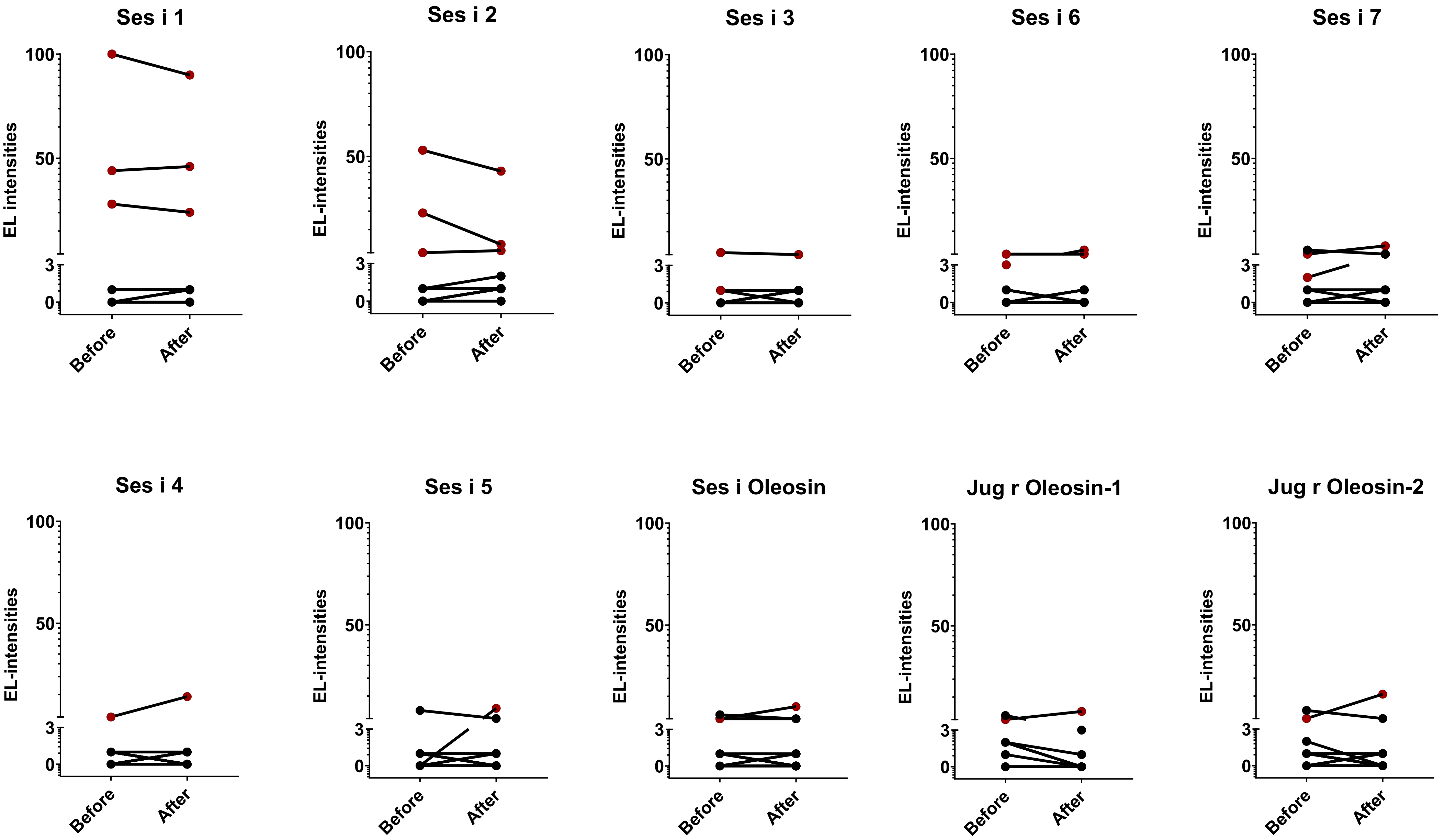

Figure S3: Sera with sIgE levels greater than an EUROLINE (EL-) intensity of 3 were marked accordingly to their study group (green: group 1a – sesame allergic without sensitisation, red: groub 1b – sesame allergic with sensitisation, black: group 2 – sesame tolerant with sensitisation).
